# The effect of probiotics on surgical outcomes in patients with gastric cancer: a meta-analysis of randomized controlled trials

**DOI:** 10.3389/fsurg.2023.1254597

**Published:** 2023-10-13

**Authors:** Wei Ye, Bo Dong, Guanglin Li, Yuqiang Zhang

**Affiliations:** Department of General Surgery, People’s Hospital of Rongchang District, Chongqing, China

**Keywords:** probiotics, gastric cancer, infectious complications, clinical outcomes, meta-analysis

## Abstract

The effect of probiotics on postoperative infectious complications and nutritional status in patients with gastric cancer is still controversial, and a comprehensive search and analysis of the current relevant evidence is necessary. Our study aimed to define the effects of probiotics on surgical outcomes in gastric cancer patients undergoing surgery. Up to June 20, 2023, Embase, PubMed, Web of Science, and Cochrane databases were searched for randomized controlled trials of probiotics in gastric cancer patients undergoing surgery. Relative risk (RR) or mean difference (MD) was used to calculate the effect sizes using RevMan 5.3. A total of nine studies reporting on 861 participants were included. Perioperative supplementation with probiotics did not improve weight loss (MD 0.73 kg; 95% CI: −0.56, 2.02) or serum prealbumin levels (MD 9.48 mg/L 95% CI: −3.43, 22.40), but did reduce the incidence of postoperative infectious complications (RR 0.46, 95% CI 0.28, 0.77), shorten the time to first exhaust (MD −11.27 h; 95% CI: −16.83, −5.70), the time to first defecation (MD −15.71 h; 95% CI: −25.62, −5.79), and the length of hospital stay (MD −0.94 days; 95% CI: −1.33, −0.55), and increase serum albumin levels (MD 0.73 g/L; 95% CI: 0.01, 1.46) in gastric cancer patients undergoing surgery. Probiotics are effective in preventing postoperative infectious complications, promoting postoperative recovery, and improving nutritional status in gastric cancer patients undergoing surgery. Our study highlights the importance of probiotics for healthcare systems and offers a potential strategy to improve the prognosis and reduce the medical burden of gastric cancer patients undergoing surgery.

## 1. Introduction

Gastric cancer is one of the most common malignancies worldwide and the fourth most common cause of cancer-related death, with more than 1,000,000 new cases diagnosed each year (1). In the United States and Europe, the five-year survival rate for advanced gastric cancer is less than 30 percent (2). Surgical resection is considered to be the most effective treatment for gastric cancer. In recent years, although many adjuvant therapies have been proposed, the postoperative morbidity and mortality of gastric cancer are still significant (3). Postoperative morbidity of gastric cancer has been reported to range from 1.88% to 59.8% (4), and a recent large national study in Germany showed that the in-hospital mortality after elective gastric cancer surgery was as high as 6.2% (5). Postoperative complications of gastric cancer surgery can lead to longer hospitalization days and increased hospitalization costs, among which intestinal obstruction is a common postoperative complication of gastric cancer (3). In addition, patients with gastric cancer are often accompanied by severe malnutrition (6), which can also lead to increased postoperative morbidity and mortality, and even affect the overall survival of patients (2). How to promote postoperative recovery of intestinal function, improve the nutritional status of patients, and reduce postoperative complications in patients with gastric cancer has become a focus of extensive attention by researchers.

Gastric surgery typically destroys the gastrointestinal mucosal barrier, resulting in increased permeability of the gastrointestinal mucosa and disorders of intestinal flora, and damage to the normal immune system (7). Gut microflora is a potential target for improving perioperative outcomes of gastric cancer. Probiotics are a group of living microbes that are beneficial when consumed in moderate amounts (8). Probiotics have been widely used to treat a variety of gastrointestinal diseases due to their extensive beneficial activities such as anti-inflammatory, anti-tumor, and immunomodulatory effects (9–13). Probiotics have shown great potential in preventing complications from intestinal surgery (14). The meta-analysis by Chowdhury et al. showed that probiotics can effectively reduce the incidence of complications such as postoperative infection in abdominal surgery (15). Probiotics could also promote recovery of intestinal function in patients undergoing colorectal surgery (16). However, relatively fewer studies explored the effect of probiotics on gastric cancer surgery, and there are inconsistent results among the studies. In addition, there is a lack of meta-analyses to summarize the current inconsistent evidence.

We hypothesized that probiotic supplementation would reduce postoperative infectious complications, promote postoperative recovery, and improve nutritional status. Here, we conducted a comprehensive search of relevant clinical studies and conducted a meta-analysis to explore the impact of probiotics supplementation on gastric cancer surgery.

## 2. Methods

### 2.1 Search strategy

This meta-analysis was successfully registered on PROSPERO (registration no. CRD42023440075). A systematic search for the current meta-analysis was conducted on material published from inception, to June 20, 2023, in the Cochrane Library, EMBASE, PubMed, and the Web of Science databases, using the following search string: (Gastric Neoplasm OR Stomach Neoplasm OR Gastric Cancer OR Stomach Cancer OR Gastric Tumor OR Stomach Tumor OR Gastric Carcinoma OR Stomach Carcinoma) AND (probiotics OR probiotic OR Bifidobacterium OR Lactobacillus OR Enterococcus OR Limosilactobacillus) (Table 1). No language restrictions were imposed. The list of references for the relevant reviews and included studies were also searched.

**Table 1:** Electronic search strategy.

Database	Search term (establish to June 20, 2023)	Number
PubMed (all fields)	#1: Gastric Neoplasm OR Stomach Neoplasm OR Gastric Cancer OR Stomach Cancer OR Gastric Tumor OR Stomach Tumor OR Gastric Carcinoma OR Stomach Carcinoma	#1: 180,577
	#2: probiotics OR probiotic OR Bifidobacterium OR Lactobacillus OR Enterococcus OR Limosilactobacillus	#2: 113,254
	#3: #1 AND #2	#3: 479
Embase (all fields)	#1: Gastric Neoplasm OR Stomach Neoplasm OR Gastric Cancer OR Stomach Cancer OR Gastric Tumor OR Stomach Tumor OR Gastric Carcinoma OR Stomach Carcinoma	#1: 184,256
	#2: probiotics OR probiotic OR Bifidobacterium OR Lactobacillus OR Enterococcus OR Limosilactobacillus	#2: 174,684
	#3: #1 AND #2	#3: 808
Cochrane library trials (all fields)	#1: Gastric Neoplasm OR Stomach Neoplasm OR Gastric Cancer OR Stomach Cancer OR Gastric Tumor OR Stomach Tumor OR Gastric Carcinoma OR Stomach Carcinoma	#1: 12,061
	#2: probiotics OR probiotic OR Bifidobacterium OR Lactobacillus OR Enterococcus OR Limosilactobacillus	#2: 12,883
	#3: #1 AND #2	#3: 94
Web of science (all fields)	#1: Gastric Neoplasm OR Stomach Neoplasm OR Gastric Cancer OR Stomach Cancer OR Gastric Tumor OR Stomach Tumor OR Gastric Carcinoma OR Stomach Carcinoma	#1: 166,232
	#2: probiotics OR probiotic OR Bifidobacterium OR Lactobacillus OR Enterococcus OR Limosilactobacillus	#2: 145,321
	#3: #1 and #2	#3: 646

### 2.2. Study selection

Inclusions contain: (1) randomized controlled trials (RCTs) (2) intervention with probiotics (any species, dose, and strain), (3) comparing with standard diet or placebo, (4) patients with gastric cancer who underwent operation (5) the outcomes included any of the following: the time to first exhaust, weight, serum prealbumin level, serum albumin level, the time to first defecation, length of hospital stay, and the incidence of postoperative complications. Primary outcome indicator was the incidence of postoperative infectious complications, and the secondary outcome were the time to first exhaust, weight, serum prealbumin level, serum albumin level, the time to first defecation, and length of hospital stay.

Exclusions contain: review, non-randomized studies, conference abstracts, case reports, letters to the editor, technical reports, and non-human studies.

### 2.3. Data extraction

Data, including study type, type of surgery, country, sample size, age, diagnosis, sex, the first author, year of publication, intervention type, control groups, number of treatment days, and outcomes were extracted from each study. If relevant data could not be extracted from the literature, we tried to contact the corresponding author of the study to obtain relevant information. When an outcome data was recorded at different timepoints in one study, the most recent measured data after the intervention was completed was selected as the outcome data.

### 2.4. Quality assessment

Methodological quality of all eligible trials was assessed using the Cochrane Collaboration's risk-of-bias tool (17): (1) the randomizing process, (2) allocation concealment, (3) participant and operator blinding, (4) blinding of outcome assessment, (5) incomplete data, (6) selective reporting, and (7) other biases. Two authors (Ye and Zhang) independently conducted literature retrieval, study selection, data extraction, and quality assessment of the methodology included in the study. When there were inconsistencies between the two authors, they were discussed and resolved by a third author (Ye, Dong and Zhang).

### 2.5. Statistical analysis

Relative risk (RR) with 95% confidence intervals (CI) were calculated for qualitative variables and mean difference (MD) for quantitative outcomes (17). Heterogeneity between studies was assessed using the *I*^2^ statistic (18). The random effects model was selected when *I*^2^ was >50%. Otherwise, the fixed-effect model was selected. To explore the robustness of the results, one-study exclusion test was used to examine the impact of each study on the pooled effect size. Analysis was conducted using Review 5.3 (The Nordic Cochrane Centre, The Cochrane Collaboration 2014; Copenhagen, Denmark). *P* < 0.05 was considered significant.

## 3. Results

### 3.1. Selected studies

**Figure 1:**
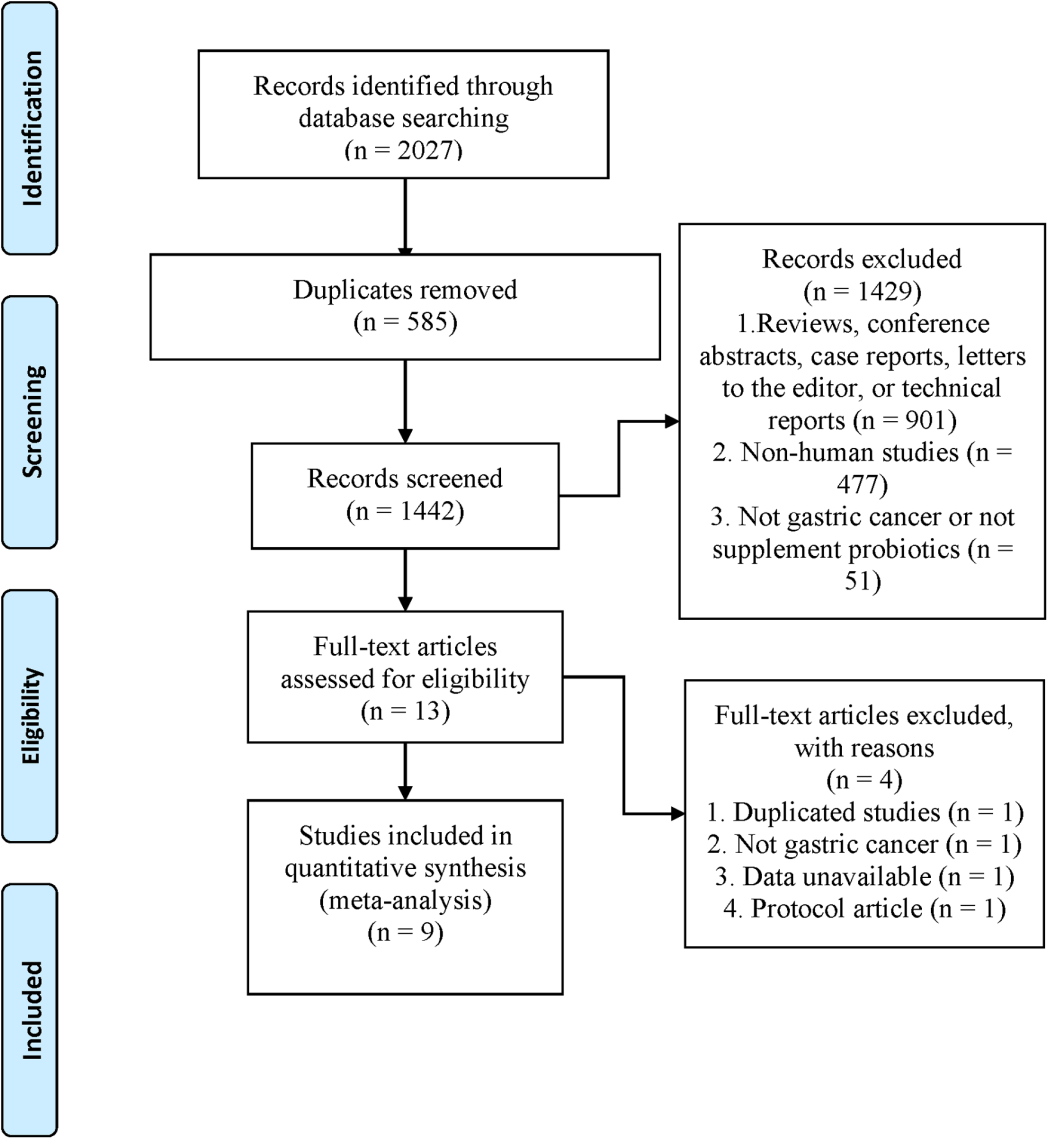
Flow chart of literature search and screening.

Our search strategy identified 2,027 records. 1,442 were retained after 585 duplicates were excluded. 1,429 articles were excluded by reading titles and abstracts (The details are summarized in Figure 1), and the remaining 13 articles were evaluated for full text. Finally, nine trials (19–27) were eligible and included for meta-analysis (Figure 1).

### 3.2. Study characteristics

Nine studies (19–27) with a total of 861 participants were published between 2006 and 2023. Eight of the studies (19, 21–27) were conducted in China and the remaining one in South Korea (20). The number of participants ranged from 60 to 140. The probiotics used in six studies (19, 21, 23–26) included *Bifidobacterium*, one (27) used *Clostridium butyricum CGMCC0313.1*, one used *Saccharomyces cerevisiae Hansen CBS 5926* (20), and one (23) did not explicitly describe the probiotic species (Table 2). The duration of intervention ranged from 7 to 28 days.

**Table 2:** Characteristics of nine eligible studies.

Study	Type of study	Sample	Age	Male	Diagnosis	Type of surgery	Intervention group	Control group	Treated days	Outcomes
Park et al. (20)	DB, RCT	I: 38 C: 38	56	55	GC	Subtotal gastrectomy and total gastrectomy	Oral *Saccharomyces cerevisiae Hansen CBS 5,926* (250 mg twice a day)	Placebo	Postoperative day 1–4 weeks	Weight, ALB
Zhao et al. (21)	RCT	I: 40 C: 40	65	40	GC	Distal gastrectomy	6 g of live *Bifidobacterium* and *Lactobacillus* in tablets (daily)	SC	Postoperative day 1–8	First flatus time; Length of hospital stay; ALB; PA
Xie et al. (22)	RCT	I: 70 C: 70	68	67	GC	Distal gastrectomy	Probiotics (three times a day)	SC	Postoperative day 1–7	Postoperative complications; The time to first flatus; Length of hospital stay; ALB; PA
Niu et al. (19)	RCT	I: 50 C: 50	53	59	GC	GC surgery	*Bifidobacterium bifidum* triple viable bacteria tablets (Mongolian Shuangqi Pharmaceutical Co., Ltd., China; S19980004) were selected, with the specification of 0.5 g/tablet. Each tablet contained no less than 0.5 × 10^7^CFU of viable bacteria. (3 tablets each time, once a day)	Placebo	Postoperative day 1–14	First defecation time; The time to first flatus
Xu et al. (23)	RCT	I: 30 C: 30	59	47	GC	Radical gastrectomy	*Bifidobacterium* and *Lactobacillus* tablets 4 g (daily)	SC	Postoperative day 1–7	Weight; The time to first flatus; Length of hospital stay; ALB; PA
Liu., (24)	DB, RCT	I: 33 C: 33	64	44	GC	Radical gastrectomy	*Bifidobacterium longum*, *Lactobacillus acidophilus*, and *Enterococcus faecalis*. Each capsule contained >1 × 10^7^CFU for each bacterial species (three capsules twice a day)	Placebo	1 week before surgery to postoperative day 7	First defecation time; The time to first flatus; postoperative complications; Length of hospital stay
Cao et al. (27)	DB, RCT	I: 47 C: 45	65	44	GC	Partial gastrectomy	Ataining contains 1.5 × 10^7^ CFU of *Clostridium butyricum* CGMCC0313.1 per gram of capsule at least (six capsules twice a day)	Placebo	Postoperative day 1–21	ALB
Liu et al. (26)	DB, RCT	I: 70 C: 70	61	112	GC	Gastrectomy	Viable *Bifidobacterium* Tablets, containing >0.5 × 106 CFU/table *B. infantis*, >0.5 × 10^6^ CFU/table *L. acidophilus*, >0.5 × 10^6^ CFU/table *Enterococcus faecalis*, >0.5 × 10^5^ CFU/table *Bacillus cereus* (three capsules three times a day)	Placebo	5–7 days before surgery	First defecation time; The time to first flatus; postoperative complications; Length of hospital stay
Zeng et al. (25)	RCT	I: 54 C: 53	56	71	GC	Partial gastrectomy	*Bifid* triple viable (0.42–0.84 g twice a day)	SC	Postoperative day 1–7	First defecation time; The time to first flatus; ALB; PA

ALB, albumin; CFU, colony forming units; C, Control group; DB, Double blind; GC, gastric cancer; I, Intervention group; PA, prealbumin; RCT, randomized controlled trial; SC, standard care.

### 3.3. Quality assessment

**Figure 2:**
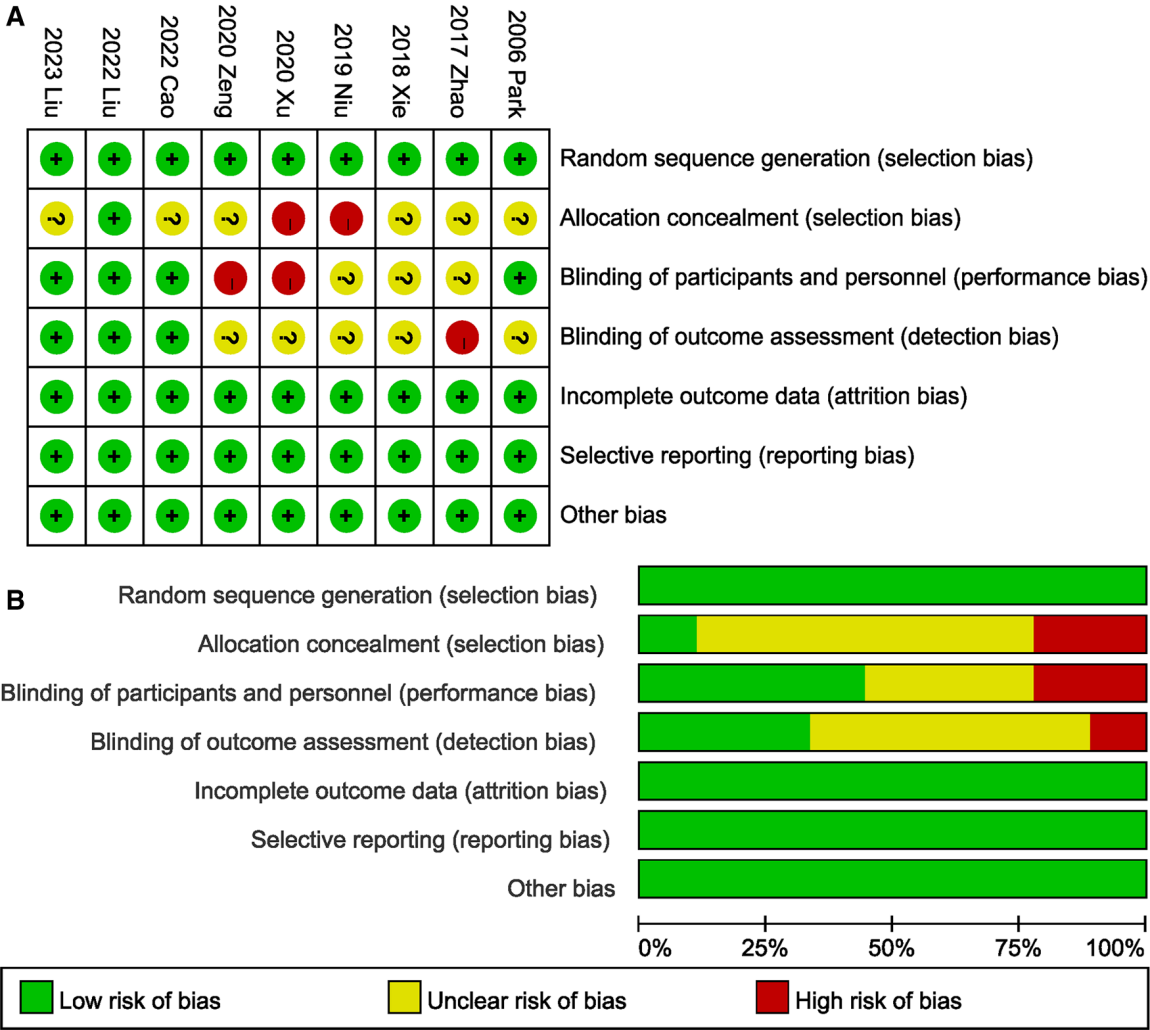
Risk of bias for each included study. (**A**) Risk of bias summary. (**B**) Risk of bias graph.

All of the trials (19–24, 26, 27) described their specific methods of randomization (Figure 2). Four tudies (20, 24, 26, 27) adopted a double-blind design, and blinded method for evaluating results were evaluated as a low bias risk in three (24, 26, 27) of the studies. The randomization scheme was appropriately hidden in one study (24). Selective reporting, incomplete outcome data, and other bias sources in all studies were evaluated as a low bias risk.

### 3.4. Meta-analysis

#### 3.4.1. Effects of probiotics on the incidence of postoperative infectious complications

**Figure 3:**
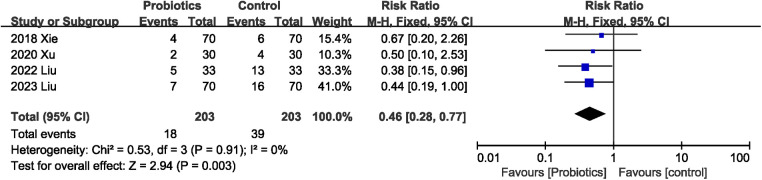
Effects of probiotics on the incidence of postoperative infectious complications.

A total of 406 participants in four studies (22–24, 26) mentioned postoperative infectious complications. The pooled effect was calculated with random-effect model. Probiotics supplementation was associated with lower risk of postoperative infectious complications (RR 0.46, 95% CI 0.28, 0.77; Heterogeneity: *I*^2^ = 0%, *P* = 0.91) (Figure 3).

#### 3.4.2. Effects of probiotics on the time to first exhaust

**Figure 4:**
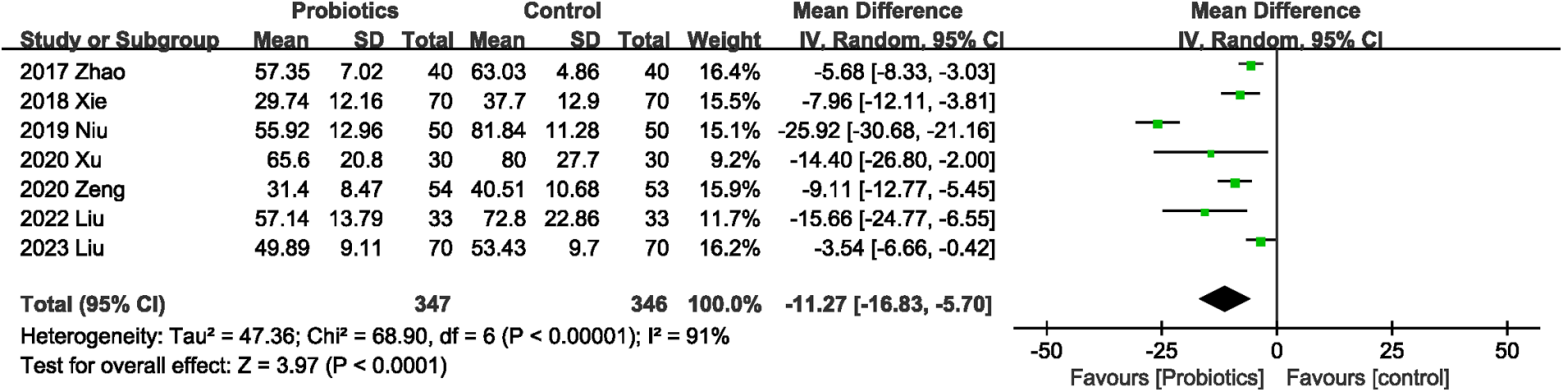
Effects of probiotics on the time to first exhaust.

A total of 693 participants in seven studies (19, 21–26) reported this outcome, all of which observed that probiotics consumption was associated with a reduction in the time to first exhaust. The overall effect indicated that probiotics could shorten the first exhaust time (MD −11.27 h; 95% CI: −16.83, −5.70), with significant heterogeneity (*I*^2^ = 91%, *P* < 0.00001) between studies (Figure 4).

#### 3.4.3. Effects of probiotics on the time to first defecation

**Figure 5:**
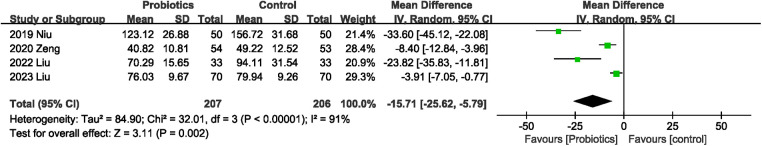
Effects of probiotics on the time to first defecation.

Four studies (19, 24–26) reported data for time to first defecation, and all showed that probiotics reduced time to first defecation. Pooled results indicated that probiotics consumption reduced the time to first defecation, with significant heterogeneity (MD −15.71 h; 95% CI: −25.62, −5.79; Heterogeneity: *I*^2^ = 91%, *P* < 0.00001) between studies (Figure 5).

#### 3.4.4. Effects of probiotics on length of hospital stay

**Figure 6:**
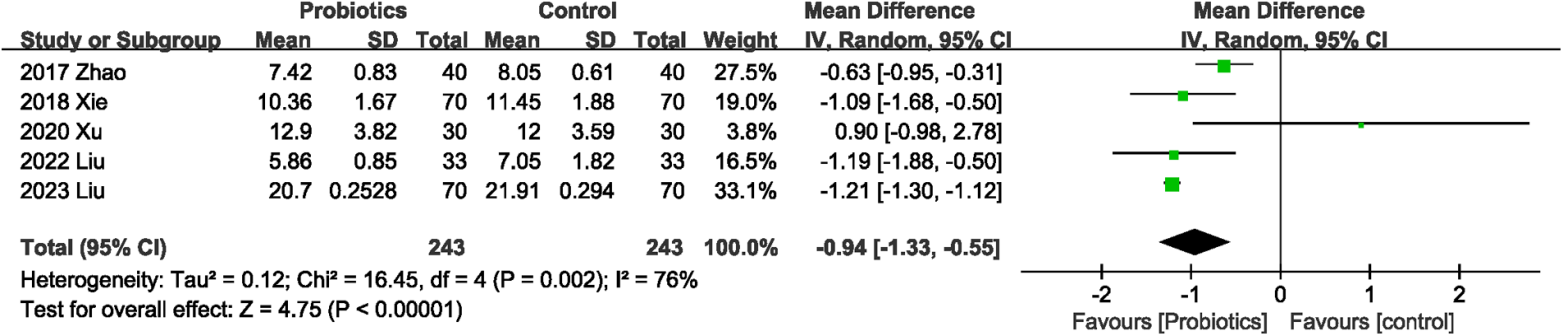
Effects of probiotics on length of hospital stay.

Length of hospital stay was mentioned in five (21–24, 26) of the nine RCTs. Four studies (21, 22, 24, 26) showed that probiotics were associated with shorter length of hospital stay, while one (23) study observed no significant difference in length of hospital stay between the probiotics and control groups. Probiotics were associated with a significant reduction in length of hospital stay (MD −0.94 days; 95% CI: −1.33, −0.55), with significant heterogeneity between studies (*I*^2^ = 76%, *P* = 0.002; Figure 6).

#### 3.4.5. Effects of probiotics on weight loss

**Figure 7:**
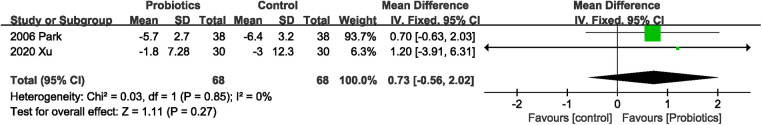
Effects of probiotics on weight loss.

The combined effect size of the two studies (20, 23) showed that probiotics did not improve weight loss (MD 0.73 kg; 95% CI: −0.56, 2.02) in patients with gastric cancer compared with controls. No significant heterogeneity (*I*^2^ = 0%, *P* = 0.85) was observed between studies (Figure 7).

#### 3.4.6. Effects of probiotics on serum albumin levels

**Figure 8:**
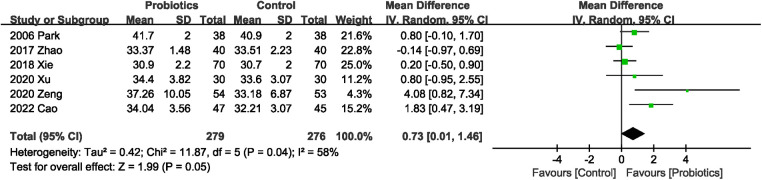
Effects of probiotics on serum albumin levels.

Six studies (20–23, 25, 27) described serum albumin levels, and probiotics supplementation significantly improved albumin levels, with high heterogeneity (MD 0.73 g/L; 95% CI: 0.01, 1.46; Heterogeneity: *I*^2^ = 58%, *P* = 0.04) between studies (Figure 8).

#### 3.4.7. Effects of probiotics on serum prealbumin levels

**Figure 9:**
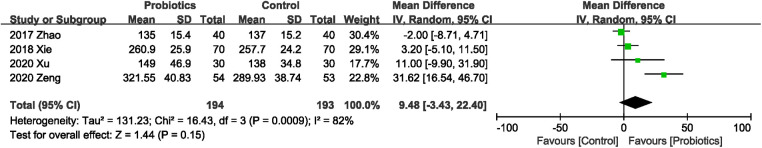
Effects of probiotics on serum prealbumin levels.

A total of 387 participants in four studies (21–23, 25) reported serum prealbumin levels, and probiotics did not increase serum prealbumin levels compared with controls, with significant heterogeneity between studies (MD 9.48 mg/L; 95% CI: −3.43, 22.40; Heterogeneity: *I*^2^ = 82%, *P* = 0.0009) (Figure 9).

### 3.5. Sensitivity analysis

The results of the sensitivity analysis showed that the total effect size of the time to first exhaust, the incidence of postoperative infectious complications, length of hospital stay, and serum prealbumin levels were not affected by the elimination of any one study. The total effect size for the time to first defecation changed when the study by Zeng et al. (25) (MD −19.86 h; 95% CI: −40.08, 0.36; Heterogeneity: *I*^2^ = 94%, *P* < 0.00001) was excluded. Sensitivity analysis indicated that Park et al.’ study (21) (MD 0.79; 95% CI: −0.14, 1.72), Cao et al.’ study (27) (MD 0.49; 95% CI: −0.20, 1.18), Zeng et al.’ study (25) (MD 0.54; 95% CI: −0.06, 1.14) and Xu et al.’ study (23) (MD 0.76; 95% CI: −0.07, 1.58) prominently affected the total effect size of serum albumin levels.

## 4. Discussion

To our knowledge, this is the first meta-analysis to evaluate the effects of probiotics on postoperative infectious complications and nutritional status in gastric cancer patients undergoing surgery. The results of this meta-analysis showed that probiotics supplementation did not improve weight loss and serum prealbumin level, but could shorten the first exhaust time, the time to first defecation, and the length of hospital stay, improve serum albumin level, and reduce the incidence of postoperative infectious complications. The results of this study have important clinical significance. Gastric cancer surgery is associated with high morbidity and mortality, and postoperative complications and poor nutritional status not only prolong the length of hospital stay and bring heavy economic burden to patients, but also are associated with poor long-term prognosis (4). The results of this study confirm the effect of probiotics on the prevention of postoperative infectious complications and the improvement of nutritional status in patients with gastric cancer. Probiotics may be a potential strategy to improve the prognosis and reduce the medical burden of gastric cancer patients undergoing surgery.

In recent years, the application of probiotics in reducing perioperative complications of abdominal surgery has become a hot spot in clinical research. Even for some highly invasive procedures, such as pancreaticoduodenectomy, probiotics are still effective (28). This is consistent with the conclusion of this study. Postoperative ileus is one of the most common complications in patients with gastric cancer, with abdominal pain, abdominal distension, weak defecation, nausea and vomiting as the main clinical manifestations, and is one of the main reasons for prolonged hospital stay (29, 30). Recovery of intestinal obstruction is fastest in the small intestine (8–12 h), followed by the stomach (1–2 days), and finally the colon (3–5 days) (30). Several randomized controlled clinical studies have shown that probiotics can promote recovery of intestinal function after abdominal surgery such as colon cancer surgery and pancreaticoduodenal surgery (16, 28, 31), similar to our results. In addition, the effect of probiotics on shortening hospital stay in patients with gastric cancer may also be related to the rapid recovery of intestinal function by probiotics.

The mechanism by which probiotics confer a benefit to gastric cancer surgery is not yet clear, which may be related to the following aspects: First, surgery leads to intestinal microflora imbalance and intestinal microflora translocation, while probiotics can protect the intestinal mucosal barrier and reduce intestinal microflora imbalance and translocation (32, 33); Second, probiotics can enhance the body’s immune function (32); Finally, probiotics can also improve intestinal motility by regulating the fermentation products of intestinal flora (31).

Malnutrition is a common and painful problem for people with gastric cancer, which can be exacerbated by gastric cancer surgery and stress (34, 35). Malnutrition is one of the damaging factors of immune function and is closely related to the increase of hospital stay and mortality (35, 36). Weight loss alone or in combination with laboratory parameters such as albumin and prealbumin is a major indicator of malnutrition (35). Therefore, the effects of probiotics on weight loss, serum albumin and serum prealbumin in gastric cancer patients undergoing surgery were analyzed in this study. Albumin is an important predictor of short-term complications after gastric cancer surgery (37). Our meta-analysis showed that probiotics were associated with improved serum albumin levels. Similarly, Zheng et al. (38) found that supplementation with *Bifidobacterium tetravaccine* tablets seven days after partial gastrectomy significantly improved nutritional parameters, including albumin and total protein. The combined results showed that probiotics did not reduce weight loss, but all the included studies (20, 23) showed a trend of improvement in weight loss, which may be related to the small number of studies included. More trials are needed in the future to explore the effect of probiotics on weight loss in gastric cancer patients undergoing surgery.

Several strength points warrant mention: on the one hand, only RCTs were included, which improves the reliability of the conclusions of our study. On the other hand, we conducted extensive literature search (besides database retrieval, hand search was used).

There are several limitations in our meta-analysis. First, only nine trials were included and included some trials with a small sample size. Secondly, there is a large heterogeneity in some outcome indicators in this study, which may be related to the differences in the species, dose and length of treatment of probiotics used between studies. However, due to the limited number of studies included, we were unable to perform subgroup analysis and meta-regression. In addition, some outcome measures, such as the time to first defecation and serum albumin levels, are not robust enough, and more studies are needed to further explore the effects of probiotics on these indicators. Finally, only four of the studies included had a double-blind design.

In conclusion, our study shows that probiotics supplementation can effectively prevent postoperative infectious complications, promote postoperative recovery, and improve nutritional status in patients undergoing gastric cancer surgery. But given some limitations, these results need to be interpreted with caution. Large, high-quality prospective studies are needed to verify the benefits of probiotics in gastric cancer patients undergoing surgery.
